# Clinical Outcomes of Critically Ill Patients Using Inhaled Nitric Oxide (iNO) during Intrahospital Transport

**DOI:** 10.1155/2021/6633210

**Published:** 2021-05-05

**Authors:** Leonid Koyfman, Omri Simchon, Anna Koyfman, Shoshana Mushaev, Benjamin F. Gruenbaum, Ron Gal, Michael Friger, Natan Arotsker, Alexander Zlotnik, Moti Klein, Evgeni Brotfain

**Affiliations:** ^1^Department of Anesthesiology and Critical Care, General Intensive Care Unit, Soroka Medical Center, Ben-Gurion University of the Negev, Beer Sheva, Israel; ^2^Department of Radiology, Meir Medical Center, Kfar Saba, Israel; ^3^Department of Internal Medicine, Soroka Medical Center, Ben-Gurion University of the Negev, Beer Sheva, Israel; ^4^Department of Anesthesiology and Perioperative Medicine, Mayo Clinic, Jacksonville, FL, USA; ^5^Department of Public Health, Faculty of Health Sciences, Ben-Gurion University of the Negev, Beer Sheva, Israel

## Abstract

Critically ill patients with severe hypoxemia are often treated in the intensive care unit (ICU) with inhaled nitric oxide (iNO). These patients are at higher risk when they require intrahospital transportation. In this study, we collected clinical and laboratory data from 221 patients who were hospitalized in the general ICU and treated with iNO at Soroka Medical Center, Israel, between January 2010 and December 2019. We retrospectively compared the 65 patients who received iNO during intrahospital transportation to the 156 patients who received iNO without transportation. Among critically ill patients who were transported while being administered iNO, only one patient had an adverse event (atrial fibrillation) on transport. We found that maximal iNO dosage during ICU stay, duration of mechanical ventilation, and percent of vasopressor support were the only independent risk factors for ICU mortality in both study groups. No difference in primary outcome of ICU mortality rate was found between the critically ill patients treated with iNO during intrahospital transportation and those who were treated with iNO but not transported during the ICU stay. We anticipate that this study will advise clinical decision-making in the ICU, especially when treating patients who are administered iNO.

## 1. Introduction

It is widely known that patients in the intensive care unit (ICU) are at increased risk for complications when transported within the hospital for diagnostic or therapeutic procedures [1–3]. Parmentier-Decrucq et al. [3] recorded and analyzed 262 intrahospital transportations of 184 patients who needed a computed tomography (CT) scan. A total of 45.8% of the patients suffered from complications and 16.8% of the patients suffered major complications, including oxygen desaturation, extubation, hemodynamic instability, and increased vasopressor dose. Out of all the complications, 16.8% were patient-related and 32.8% were equipment-related.

Critically ill patients with severe hypoxemia, often caused by acute respiratory distress syndrome (ARDS), are at particular risk from complications of hospital transportation. These patients require advanced mechanical ventilation with a high mean airway pressure and positive end-expiratory pressure (PEEP), a high percentage of oxygen, deep sedation, and muscle relaxation [4, 5]. Some of these severely affected patients are commonly treated with inhaled nitric oxide (iNO).

A large percentage of ICU patients treated with iNO develop rebound hypoxemia and pulmonary hypertension following immediate withdrawal of iNO. Thus, when discontinuing iNO, it is important to slowly wean patients off the gas [1]. Any transportation of this patient subpopulation outside of the ICU is complex and potentially life-threatening as interruption of iNO delivery, for even a short period of time, can be dangerous [1].

Despite the common use of iNO in intensive care units throughout the world, few reports have examined the use of continuous iNO therapy in critically ill patients during intrahospital transportation while in the ICU [6–8]. In previously published data, there have been reports of continuous iNO for transfer of adult critical care patients with severe hypoxemia to a tertiary center [4]. Flatten et al. presented a case report of a critically ill patient with severe ARDS who successfully underwent intrahospital transport and major surgery while being treated with iNO [9]. Also, several studies describe successful usage of iNO during interhospital transport of preterm and full-term newborns suffering from persistent pulmonary hypertension [10–12]. We retrospectively assessed and compared the clinical outcomes of critically ill patients who were treated with iNO during their ICU stay and during intrahospital transportation to the outcomes of patients who received iNO only during their ICU stay.

## 2. Methods

The study was conducted at Soroka Medical Center, a 1,000-bed tertiary care university hospital located in Israel. We retrospectively collected the clinical and laboratory data of all the critically ill patients who were treated with iNO while hospitalized in the general ICU at Soroka between January 2010 and December 2019. Clinical information was retrieved from our computerized Registration Information System (MetaVision®, iMDsoft® Israel and “OFEK” Electronic Data). The study was approved by the Human Research and Ethics Committee at Soroka University Medical Center (RN -0237-19-SOR).

### 2.1. Inclusion Criteria

All critically ill patients aged ≥18 years who were hospitalized for more than 72 hours in the ICU at Soroka Medical Center between January 2010 and December 2019 and who were treated with iNO during their ICU stay were eligible for inclusion in the study.

### 2.2. Exclusion Criteria

The following patients were excluded from the study: critically ill patients who were hospitalized in preparation for organ donation and patients whose medical records contained insufficient data.

### 2.3. Variables and Measures

The demographic data included the patients' admission diagnoses, the presence or absence of comorbidities, previous chronic drug treatment, and the duration of the patients' ICU and hospital admissions. The following laboratory findings were documented at the time of admission to the ICU: hemoglobin level and white blood cell count, as well as blood glucose, urea, and creatinine levels. The following parameters were also recorded: length of mechanical ventilation (MV); ventilation parameters such as PaO_2_/FiO_2_ ratio, PEEP, FiO_2_, and lung compliance; the eventual success or failure of weaning from MV and days without MV (ventilator-free days); use of vasopressors and steroids during ICU hospitalization; parameters related to the treatment of iNO such as length of iNO treatment and iNO dose; parameters related to iNO intrahospital transport including the frequency of transport during the ICU hospitalization period, iNO dose during transport, place of transport, duration of transport, and the presence or absence of complications during transport and their type; echocardiographic features such as left and right ventricle function or evidence of pulmonary hypertension; Acute Physiology and Chronic Health Evaluation-II (APACHE-II) and Therapeutic Intervention Scoring System (TISS) scores; and in-ICU and in-hospital mortality rates.

### 2.4. Definitions

The severity of the patients' illnesses and the presence or absence of multiorgan failure were evaluated within 24 hours of ICU admission in accordance with the patients' APACHE-II and TISS scores.

### 2.5. Study Groups

All study participants were treated with iNO. After retrospective analysis of their clinical and laboratory data, the study patients were divided into two groups: patients who were not transported outside of the ICU during their hospitalization period (Group 1) and patients that were transported outside of the ICU during iNO treatment during their hospitalization period (Group 2). Transportation was defined as any intrahospital transport of a patient outside of the ICU for any diagnostic or therapeutic procedure purposes. Intrahospital transportation included cardiorespiratory monitoring, portable mechanical ventilation machine (using similar background respiratory parameters), and mobile iNO device and tank.

### 2.6. Inhaled Nitric Oxide (iNO) Therapy

Inhaled nitric oxide (iNO) therapy was administered to mechanically ventilated, sedated, and paralyzed critically ill patients with refractory hypoxemia due to severe acute respiratory distress syndrome (ARDS) (PO_2_/FiO_2_ ratio < 100, FiO_2_ 1.0, PEEP >12 cmH_2_O). The starting dose of iNO was 20 ppm.

### 2.7. Primary Endpoint

Duration of mechanical ventilation during the ICU stay was defined as the primary endpoint.

### 2.8. Secondary Endpoints

The following secondary endpoints were defined: intrahospital mortality, the duration of the patients' ICU and hospital stay, the duration of MV while in the ICU, success or failure of weaning from MV, and changes in iNO connectivity or respiratory parameters while on intrahospital transport.

### 2.9. Statistical Analysis

Statistical analysis was performed in three steps: The first step is descriptive statistics. Variables that are normally distributed are described by the mean and standard deviation (such as age, weight, and more). Variables that were normally distributed are described by median and range (e.g., time). Qualitative variables such as gender are described by their percentage distribution. The second step is univariate analysis. At this point, each variable was compared to all independent variables. Comparison of a dependent variable with quantitative variables that are normally distributed as weight/age was done by means of a *t*-test for independent samples. A comparison of a dependent variable with quantitative variables that are not normally distributed was done by means of a Mann-Whitney test. Comparison of a dependent variable with quality variables used the independent chi-squared test or Fisher's exact test as needed. The third step is multivariate mortality testing. For this testing, we used logistic regression. The model includes an independent variable and also variables that are significant in the second stage and/or clinically important. We also examined possible interactions between variables. For multivariate survival analysis, we used Cox regression. Data analysis was processed with SPSS Version 25. The significance level in multivariate analysis was set at 0.05. Data were analyzed using IBM SPSS 22 (NY, USA) and Epi Info 3.5.1 (CDC, GA, USA).

## 3. Results

A total of 221 patients received inhaled nitric oxide (iNO) while hospitalized in the general ICU during the study period. 10 patients had insufficient data records and were excluded from the study. A total of 221 study patients were divided into two groups on the basis of a retrospective analysis of their transportation outside of the ICU.

The patients' demographic, clinical, and laboratory data and the details of their ICU therapeutic management are presented in Table 1. There were no significant differences between the two groups in their demographic parameters (age, weight, and gender), admission diagnoses, APACHE and TISS scores, or their laboratory data (Table 1). The proportion of patients with a past history of chronic obstructive pulmonary disease (COPD) and chronic hypertension (HTN) was significantly higher in Group 2 (transported patients) than in Group 1 (nontransported patients) (*PP*=0.02, 0.04, respectively, Table 1). The percentages of patients treated with vasopressor support or steroid treatment or who had abnormal echocardiography findings were not different between both study groups. All patients included in the present study were sedated, continuously paralyzed with muscle relaxants, and mechanically ventilated. Basic ventilation parameters including PaO_2_/FiO_2_ ratio, PEEP, FiO_2_, and lung compliance were similar in both study groups.

Analysis of the patients' iNO treatment data during the ICU stay demonstrated a significant difference in maximal iNO dosage and iNO length of treatment between the two study groups, with the Group 2 transported patients consistently showing higher iNO dose and longer iNO treatment than the Group 1 nontransported patients (*P* < 0.017 and <0.0001, respectively, Table 2). Critically ill patients treated by iNO (Group (2) were transported during their ICU stay at least once (71.6% of patients) to CT imaging and OR in the majority of cases (Table 2). Overall time of intrahospital transportation was 116.89 ± 10.7 minutes. One of these patients (Group 2, transported patients) developed rapid atrial fibrillation (AF) during intrahospital transportation (heart rate > 100 beats/min) which was treated successfully by continuous amiodarone.

Routine chest X-rays after comparison to X-rays before intrahospital transportation of critically ill patients with iNO demonstrated no change in 30% of patients, while 25% had worse outcomes in radiologic criteria of ARDS. 35% of chest X-Rays showed radiographic improvement after intrahospital transportation. No further clinical deterioration or adverse effects were demonstrated after intrahospital transport.

The ICU and intrahospital mortality rates were similar in both groups (Table 3). There were no differences between the groups in MV, duration of their ICU stay and hospital stay, proportion of patients weaned successfully from MV, and the number of ventilation-free days (Table 3).

Table 4 shows the results of multivariate logistic regression analysis of Group 1 and Group 2. Maximal iNO dosage during ICU stay, duration of mechanical ventilation, and percent of vasopressor support were found to be the only independent risk factors for ICU mortality in both study groups.

## 4. Discussion

Nitric oxide (NO) is a selective pulmonary vasodilator, and it has been successfully used as a rescue therapy for severe refractory hypoxemia [7]. iNO administration reduces endogenous NO production, and therefore rapid withdrawal of iNO can cause a significant rebound pulmonary hypertension and subsequent hypoxemia. This can be avoided by gradual withdrawal [7, 8]. iNO is administered most commonly to invasively ventilated patients.

Despite the clear clinical benefit for the improvement of oxygenation and minimal potential adverse events related to iNO use in critically ill patients with ARDS, there is insufficient evidence that it improves patients' clinical outcome [8]. Furthermore, the difficulties arising from equipment and clinical management of critically ill patients requiring diagnostic procedures, urgent surgeries, or intrahospital relocations are associated with significant complications [2].

In the present study, we compared the clinical outcomes of two groups of critically ill patients who were treated by iNO during the ICU stay. Group 1 consisted of patients who were not transported outside of the ICU during their hospitalization period. Group 2 consisted of patients who were transported outside of the ICU while being treated with iNO during their hospitalization period. The Group 2 patients (iNO transportation group) demonstrated significantly higher iNO doses and longer iNO treatment compared to Group 1 patients (nontransported patients). Overall intrahospital transportation of critically ill patients with iNO was safe (with only one case of rapid AF) and average time of transport outside the ICU with iNO was about two hours. Moreover, 15% and 4.5% of critically ill patients were transported safely with iNO two and three times, respectively, during the ICU stay. Our findings are similar to those found in the neonatal population in newborns with severe hypoxemia refractory to conventional therapy due to congenital diaphragmatic hernia (CDH), PPHN, meconium aspiration syndrome, sepsis, and non-CDH pulmonary hypoplasia [12]. Kinsella et al. [12] observed uneventful and safe interhospital iNO transport for 25 term newborns with overall iNO dosage at about 25 ppm. Teman et al. [4] demonstrated significant improvement in oxygenation of patients with severe hypoxemia using iNO during safe transport to a tertiary care center. In the present study, the patients were treated with an even higher iNO dose (>30 ppm) during intrahospital transport.

Several recent studies have shown the beneficent perioperative use of iNO for cardiac surgery, as well as lung and cardiac transplantations [6]. Two studies by Flaatten et al. [9] and Haddad et al. [13] reported the safety of iNO use during different types of surgery for adult and pediatric populations. In the data presented here, about 42% of critically ill patients were safely transported to the OR and used iNO during surgery.

Importantly, our univariate statistical analysis did not show differences in the ICU or hospital mortality rates between the two subpopulations. Moreover, patients in both study groups had similar durations of MV, vasopressor support, ICU, and hospital stay. Furthermore, multivariate analysis demonstrated that the following factors were independent predictors of increased ICU mortality among both study groups: maximal iNO dosage during ICU stay, duration of mechanical ventilation, and percent of successful weaning from mechanical ventilation. There is not sufficient evidence in recently published literature to support that iNO reduces mortality in critically ill patients with severe ARDS [7, 8].

This study has several limitations, including a lack of adequate information concerning the patients' medical history. In addition, our data did not contain complete records of complications that may have taken place after intrahospital transport of severe ARDS patients treated by iNO. Furthermore, the groups that were examined were not identical and had different underlying conditions. These differences can significantly impact outcome. We anticipate that future prospective studies on this topic will correct any limitations caused by this study's retrospective design.

In the present study, we demonstrated that maximal iNO dosage during ICU stay is associated with high risk of ICU mortality in critically ill patients with severe ARDS. We suggest that this finding is correlated with severe critical illness of ARDS patients hospitalized in the ICU rather than an effect of iNO treatment on patients' clinical outcome. Further studies are needed to better understand the clinical significance of iNO treatment use during intrahospital and interhospital transportation in critically ill patients.

## 5. Conclusion

No difference in primary outcome (ICU mortality rate) was found between the ICU patients treated with iNO with intrahospital transportation and those who were not transported during the ICU stay. This study provides an insight into the utility of iNO for critically ill patients, suggesting that it does not primarily change outcomes even for patients who undergo intrahospital transportation. It might be inferred that intrahospital transfer with iNO is safe for the critically ill population. We anticipate the relevance for this study in clinical decision-making within the ICU, especially for treating critically ill patients receiving iNO.

## Figures and Tables

**Table 1:** The demographics, underlying conditions, admission diagnoses, laboratory data, and therapeutic management during the ICU stay.

	Group 1^*∗*^ (*n* = 156)	Group 2^*∗*^ (*n* = 65)	*P* value^∗∗^
Age, years (mean ± SD^b^)	50.92 ± 19.826	50 ± 18.822	0.751
Weight, kg (mean ± SD)	80.763	90.523	0.655
Male gender, no. (%)	112/156 (72.4%)	47/65 (72.3%)	0.985
Admission diagnosis, no. (%)^∗∗∗^			
Sepsis	47/156 (30.1%)	28/65 (43.1%)	0.174
Multiple traumas	67/156 (43.6%)	24/65 (36.9%)	0.179
Other	41/156 (26.3%)	13/65 (20%)	0.97
ICU scores at admission (units, mean ± SD)			
APACHE^c^ score	26.49 ± 4.7	25.8 ± 4.28	0.34
TISS^d^ score	29.81 ± 9.51	27.57 ± 5.54	0.07
Laboratory data during iNO treatment in ICU			
WBC^e^ (1000 cells/*μ*L, mean ± SD)	16.582 ± 9.523	14.986 ± 8.652	0.245
Hemoglobin (g\dL)	11.528 ± 3.033	11.420 ± 2.586	0.801
Serum urea (mmol/L)	58.32 ± 53.086	54.29 ± 43.592	0.590
Serum creatinine (mmol/L)	1.626 ± 2.892	1.286 ± 0.903	0.354
Blood glucose level (mg/dL)	156.79 ± 62.036	161.85 ± 61.739	0.581
Underlying conditions, no. (%)			
CIHD^f^	36/156 (23.1%)	13/65 (20.1%)	0.83
COPD^g^	24/156 (15.4%)	20/65 (30.8%)	0.02
HTN^h^	31/156 (19.9%)	18/65 (27.7%)	0.04
Other^i^	64/156 (41.7%)	14/65 (21.5%)	0.03
Therapeutic management and diagnostic tests during ICU stay, no. (%)^∗∗∗∗^			
Vasopressor support	119/156 (76.3%)	46/65 (69.2%)	0.275
Steroid treatment	87/156 (56.4%)	40/65 (61.5%)	0.482
ECHO study findings			
Left ventricle dysfunction (moderate/severe)	47/156 (30.1%)	20/65 (29.7%)	0.297
Right ventricle dysfunction	50/156 (32%)	18/65 (28%)	0.616
Pulmonary hypertension (moderate/severe)	62/156 (40.3%)	21/65 (33.3%)	0.757

^*∗*^Group 1: critically ill patients who were not transported outside of the ICU during their hospitalization period. Group 2: critically ill patients who were transported outside of the ICU during iNO treatment during their hospitalization period. ^∗∗^Data are considered statistically significant if *P* < 0.05. ^∗∗∗^Other diagnoses include intracerebral hemorrhage, cardiogenic shock, eclampsia, SAH, NSTEMI, and pulmonary edema. ^∗∗∗∗^Vasopressor support: norepinephrine continuous infusion; steroid treatment: concomitant hydrocortisone administration. ^a^ICU: intensive care unit. ^b^SD: standard deviation. ^c^APACHE: Acute Physiology and Chronic Health Evaluation on admission to ICU.^. d^TISS: Therapeutic Intervention Scoring System on admission to ICU. ^e^WBC: white blood cells. ^f^CIHD: chronic ischemic heart disease. ^g^COPD: chronic obstructive pulmonary disease. ^h^HTN: chronic hypertension. ^I^Other: diabetes mellitus II, dyslipidemia, alcohol abuse, fat embolism, and pulmonary embolism.

**Table 2:** iNO treatment data and intrahospital patient transportation (Group (2)) data during the ICU stay.

	Group 1^*∗*^ (*n* = 156)	Group 2^*∗*^ (*n* = 65)	*P* value^∗∗^
Maximal iNO dosage during ICU stay (ppm, mean ± SD)	35.08 ± 15.618	40.95 ± 18.488	0.017
iNO dosage during intrahospital transportation (ppm, mean ± SD)	NA	32.51 ± 14.181	—
iNO treatment length (days, mean ± SD)	4.5 ± 4.643	9.57 ± 10.239	0.0001
Duration of intrahospital transportation (minutes, mean ± SD)	NA	116.89 ± 10.7	—
Intrahospital transport location (%)			
CT^a^	NA	52.2%	—
Operating room (OR)	NA	41.8%	—
Other (angiography, other intensive care unit)	NA	6%	—
Frequency of intrahospital transportation (%)			
One	NA	71.6%	—
Two	NA	14.9%	—
Three	NA	4.5%	—

^*∗*^Group 1: critically ill patients who were not transported outside of the ICU during their hospitalization period. Group 2: critically ill patients who were transported outside of the ICU during iNO treatment during their hospitalization period. ^∗∗^Data are considered statistically significant if *P* < 0.05.^a^CT: computerized tomography.

**Table 3:** Clinical outcome endpoints of both study groups (mean ± SD and median).

	Group 1^*∗*^ (*n* = 156)	Group 2^*∗*^ (*n* = 65)	*P* value^∗∗^
Duration of MV^a^ during ICU^b^ stay (days, mean ± SD)	18 (2–48)	24 (1–62)	0.078
Days without MV (ventilation-free days) (days, mean ± SD)	2.81 ± 3.591	2.95 ± 4.488	0.799
Patients weaned from MV, no. (%)	62.2%	63.1%	0.513
Length of ICU stay (days, mean ± SD)	22 (5–52)	27 (4–66)	0.108
Length of hospital stay (days, mean ± SD)	32.5 (11–75)	40 (9–82)	0.31
ICU mortality (%)	32.1%	29.2%	0.68
Intrahospital mortality (after discharge from ICU)			
(%)	0	1.9%	0.26

^*∗*^Group 1: critically ill patients who were not transported outside of the ICU during their hospitalization period. Group 2: critically ill patients who were transported outside of the ICU during iNO treatment during their hospitalization period ^∗∗^Data are considered statistically significant if *P* < 0.05. ^a^MV: mechanical ventilation. ^b^ICU: intensive care unit.

**Table 4:** Multivariate logistic regression analysis for risk factors for ICU mortality in critically ill patients with iNO treatment.

	OR^a^	95% CI^b^ of OR	*P* value
Maximal iNO dosage during ICU^c^ stay (ppm, mean ± SD)	1.042	1.009–1.077	0.013
Duration of MV^d^ during ICU stay (days, mean ± SD)	0.932	0.895–0.97	0.001
Vasopressors support (mean ± SD)	64.01	2.095–195.5	0.017

^a^OR: odds ratio. ^b^CI: confidence interval. ^c^ICU: intensive care unit. ^d^MV: mechanical ventilation.

## Data Availability

The data that support the findings of this study are available from the corresponding author, Evgeni Brotfain, upon reasonable request.
