# Corrigendum to “Clinical Outcomes of Critically Ill Patients Using Inhaled Nitric Oxide (iNO) during Intrahospital Transport”

**DOI:** 10.1155/2021/9794018

**Published:** 2021-06-16

**Authors:** Leonid Koyfman, Omri Simchon, Anna Koyfman, Shoshana Moshinsky, Benjamin Fredrick Gruenbaum, Ron Gal, Michael Friger, Natan Arotsker, Alexander Zlotnik, Moti Klein, Evgeni Brotfain

**Affiliations:** ^1^Department of Anesthesiology and Critical Care, General Intensive Care Unit, Soroka Medical Center, Ben-Gurion University of the Negev, Beer Sheva, Israel; ^2^Department of Radiology, Meir Medical Center, Kfar Saba, Israel; ^3^Department of Internal Medicine, Soroka Medical Center, Ben-Gurion University of the Negev, Beer Sheva, Israel; ^4^Department of Anesthesiology and Perioperative Medicine, Mayo Clinic, Jacksonville, FL, USA; ^5^Department of Public Health, Faculty of Health Sciences, Ben-Gurion University of the Negev, Beer Sheva, Israel

In the article titled “Clinical Outcomes of Critically Ill Patients Using Inhaled Nitric Oxide (iNO) during Intrahospital Transport” [1], there was a spell error in author Shoshana Moshinsky's name in the author list, where “Shoshana Mushaev” should have read “Shoshana Moshinsky.” This is corrected as shown in the author details above.
